# Habitat type and ambient temperature contribute to bill morphology

**DOI:** 10.1002/ece3.911

**Published:** 2014-02-13

**Authors:** David Luther, Russell Greenberg

**Affiliations:** 1 Biology Program, George Mason UniversityFairfax, Virginia, 22030; 2 Smithsonian Migratory Bird Center, Smithsonian Conservation Biology InstituteNational Zoological Park, Washington, District of Columbia, 20008

**Keywords:** Ambient temperature regulation, bill morphology, island syndrome, mangrove, sexual dimorphism, yellow warbler.

## Abstract

Avian bills are iconic structures for the study of ecology and evolution, with hypotheses about the morphological structure of bills dating back to Darwin. Several ecological and physiological hypotheses have been developed to explain the evolution of the morphology of bill shape. Here, we test some of these hypotheses such as the role of habitat, ambient temperature, body size, intraspecific competition, and ecological release on the evolution of bill morphology. Bill morphology and tarsus length were measured from museum specimens of yellow warblers, and grouped by habitat type, sex, and subspecies. We calculated the mean maximum daily temperature for the month of July, the hottest month for breeding specimens at each collecting location. Analysis of covariance models predicted total bill surface area as a function of sex, habitat type, body size, and temperature, and model selection techniques were used to select the best model. Habitat, mangrove forests compared with inland habitats, and climate had the largest effects on bill size. Coastal wetland habitats and island populations of yellow warblers had similar bill morphology, both of which are larger than mainland inland populations. Temperate but not tropical subspecies exhibited sexual dimorphism in bill morphology. Overall, this study provides evidence that multiple environmental factors, such as temperature and habitat, contribute to the evolution of bill morphology.

## Introduction

Birds have a wide diversity of bill shapes and sizes. Most research has attributed the diversity of bill morphology to differences in feeding ecology, both in terms of the different types of food captured but also different methods of foraging for food (Bowman 1961; Pulliam and Enders 1971; Boag and Grant 1981; Grant 1986; Smith 1990; Benkman 1993). Diet and competition are traditionally the most important factors thought to influence bill morphology (Darwin 1859; Grant 1986). Consequently, bill shape has been closely linked with ecological niches for many species such that differences between species in terms of bill shape are hypothesized to be a result of interspecific competition for food resources (Grant 1986; Temeles et al. 2000; Altshuler and Clark 2003; Grant and Grant 2006).

The ‘island syndrome’ hypothesis states that reduced interspecific and increased intraspecific competition is a potential cause of increased bill size among populations of passerines restricted to islands (Grant 1968; Blondel 2000; Scott et al. 2003). Larger bills are hypothesized to increase dietary niche breadth where selection favors trophic generalization. The island syndrome has been extended to the mainland in only two ecosystems, salt marshes and mangroves (Grenier and Greenberg 2005; Luther and Greenberg 2011), which are known to be both productive and support depauperate avifaunas. Thus, salt marshes and mangroves are ‘island like’ in that we expect similar evolutionary pressures on them as those found on islands.

Intraspecific competition can also select for greater dimorphism in bill morphology between the sexes (Selander 1966; Temeles et al. 2000, 2009). There is evidence of sexual dimorphism in bill morphology from a wide range of species such as woodpeckers, shorebirds, passerines, and more (Selander 1966; Temeles et al. 2000; Grant and Grant 2006; Greenberg and Olsen 2010; Greenberg and Danner 2013). Sexual dimorphism in bill morphology is thought to be the result of divergence in diet or foraging behavior due to competitive interactions between the sexes. Alternatively, bill dimorphism could result from sexual selection, either due to increased male–male interaction or female choice (Grant 1986).

Bird bills can also perform other essential tasks such helping to regulate avian body temperature (Scott et al. 2008; Tattersall et al. 2009; Symonds and Tattersall 2010; Greenberg and Danner 2012; Greenberg et al. 2012a,b; Greenberg and Danner 2013). Bills are appendages without insulation that have a layer of vascularized tissue, which can allow them to easily release metabolic heat (Tattersall et al. 2009; Symonds and Tattersall 2010). Bills could be ideal structures for birds to, at least partially, regulate their body temperature. It has been hypothesized that increased bill size can facilitate the dissipation of metabolic heat under hot conditions, such that birds in hotter climates should have larger bills than their counterparts in cooler climates (Greenberg and Danner 2012; Greenberg et al. 2012a,b). This mechanism may also account for bill size dimorphism, as males are the most climatically exposed sex as they patrol territories and sing from exposed perches. These hypotheses have been explored in sparrows, birds with relatively large bills for their body size, but never in birds with relatively small bills for their body size, such as warblers.

The yellow warbler (YEWA) (*Setophaga petechia*) lives throughout North and Central America from the Alaskan tundra to the mangrove forests of northern South America. There are 35 subspecies of YEWA (Lowther et al. 1999), many of which live in different habitat types and are subject to different climactic regimes. There are three supergroups of YEWA subspecies. The *aestiva* group has six subspecies, breeds throughout temperate North America in open woods and wet forest thickets and winters primarily in Central and South America. The *petechia* group has 17 subspecies and is a year-round resident in the West Indies and is largely restricted to mangrove forests. The *erithachorides* group has 12 subspecies and is a year-round resident in the mangrove forests of Central America and northern South America (Lowther et al. 1999).

Previous research indicates that bill size is greater in male YEWA that are year-round residents in mangrove forests compared with migratory YEWA that inhabit inland terrestrial habitats, even after correcting for body size (Luther and Greenberg 2011). Based on predictions from recent research on bill morphology of birds restricted to salt marshes (Greenberg and Olsen 2010; Greenberg et al. 2012a,b) and mangrove forests (Luther and Greenberg 2011), our present study examines the following four hypotheses about bill morphology in relation to differences in climate, sex, biogeography, and habitat type between populations of YEWA:

1. Based on previous findings in Luther and Greenberg (2011), in which bird populations dependent upon mangrove forests as their primary habitat had larger bills than their closest inland relatives, we hypothesize that mangrove-dependent birds will have larger bills than inland birds.
2. Based on the evidence from salt marshes where sparrow populations that are dependent upon salt marshes exhibit greater sexual dimorphism than their closest inland relatives (Greenberg and Olsen 2010), we hypothesize that birds in mangroves will exhibit greater sexual dimorphism in bill morphology than inland terrestrial birds.
3. Based on recent studies that link ambient temperature with bill size (Greenberg and Danner 2012; Greenberg et al. 2012a,b), we hypothesize that birds that inhabit regions with higher ambient temperatures will have larger bills than birds in cooler climates.
4. Based on predictions that birds in coastal wetland ecosystems have larger bills than their closest inland relatives, as well as a depauperate fauna (Greenberg and Olsen 2010), which is similar to the situation of birds with bigger bills on islands compared with their closest mainland relatives (Blondel 2000; Scott et al. 2003), we hypothesize that there will be no difference in the bill size of mangrove birds that inhabit islands and those that inhabit the mainland.

## Methods

We measured bill morphology and tarsus length of 326 yellow warbler (*S. petechia*) study skins from 13 different subspecies (see Table 1). Nine subspecies were year-round residents in mangrove habitats in Central America, South America, and the Caribbean. Four subspecies were migratory and inhabited inland terrestrial habitat in North America during their breeding season, May to August. Equal numbers of males and females were measured. We only measured North American migratory specimens collected after May 15 to ensure that they were on or in the vicinity of their breeding grounds. Measurements of mangrove subspecies were from all months of the year as they are year-round residents (Salgado-Ortiz et al. 2008). We sampled individuals throughout the species range in North America, Central America, and the Caribbean.

**Table 1:** Subspecies measured and the habitat and region in which they were collected.

Subspecies	Females	Males	Total	Habitat	Region
*S.p. rubiginosa* and *amnicola*	19	20	39	Inland	Canada and Alaska
*S.p. bartholemica, cruciana, gundlachi, rufopileata, solaris*	40	40	80	Mangrove	Caribbean
*S.p. aequatorialis, dugesi, jubaris, rufivertex, xanthotera*	39	40	79	Mangrove	Central America
*S.p. aestiva*	32	31	63	Inland	Mid-Atlantic and Southern US
*S.p. sonarana*	31	34	65	Inland	Southwest US

We used digital calipers to measure bill depth, bill width, and culmen length from the anterior edge of the nares (to the closest 0.01 mm). Tarsus length was measured from the middle of the intertarsal joint to the distal edge of the last scale before the toes (Pyle 1997) and was measured as an indicator of overall body size (Grenier and Greenberg 2005). Bill surface area was estimated with the formula for the surface area of an elliptical cone ([bill width + bill depth]/4 × bill length) (after Greenberg et al. 2012a,b). The percentage of sexual dimorphism of bill surface area was calculated ([female bill surface area−male bill surface area]/female bill surface area × 100), and values were compared between the regions (after Greenberg et al. 2012a,b). Bill and tarsus morphology measurements were square root-transformed to meet assumptions of normality.

Thirty-year monthly average temperatures were obtained from several websites for weather stations as close as possible to the collecting sites of each specimen(http://www.climate.weatheroffice.gc.ca/climate_normals/index_e.html, http://www.weather.com and http://www.climatetemp.info). For each collecting location, we calculated the mean maximum daily temperature for the month of July, the hottest month for breeding specimens, as the maximum daily temperature during the breeding season has been shown to have a stronger relationship with bill size than winter temperature (Greenberg et al. 2012a,b). July average high temperatures associated with collecting sites for YEWA in terrestrial habitats ranged from 18 to 41°C, median 31°C (Regions: Alaska/Canada 18–27°C, median 20.6°C; Mid-Atlantic/Southern US 29–33°C, median 31°C; Southwest US 30–41°C, median 34°C). YEWA in mangrove habitats had temperature ranges from 29 to 33°C, median 31°C (same range on mainland Central America and on Caribbean islands).

### Statistical analysis

We used models to predict total bill surface area as a function of sex, habitat type, and temperature. Tarsus was used as a proxy for body size (Luther and Greenberg 2011) and included in all models; thus, we did not explicitly assess the effect of body size. Tarsus length, because of its known correlation with bill size, was used as our null model. Individuals were associated with either mangrove or inland habitat types. We used analysis of variance and covariance to create the following global model and its subsets, bill surface area = sex + habitat + sex × habitat + temperature + tarsus, to assess two of the four hypotheses, 1 and 3. Bill surface area was the dependent variable, temperature, sex + tarsus, and habitat were predictor variables. Models tested include each individual variable, all combinations of variables, and the interaction between sex and habitat. We assessed Hypothesis 2, sexual dimorphism, with *t*-tests. Bonferroni was used to correct for multiple tests. For Hypothesis 4, bill size in continental compared with island populations of mangrove-dependent YEWA, we used the following global model, bill surface area = region + sex + temperature + tarsus, which was only applied to populations that inhabit mangroves. In this model, region was defined as either mainland Central America or Caribbean Islands.

Model performance was assessed using the Akaike's information criterion (AIC) corrected for sample size (AICc: Burnham and Anderson 2002), and the Δ AICc and model weights were calculated. The ratio of the model weights (evidence ratio) was used for specific comparisons. We present the adjusted R^2^ as a descriptive statistic for the models with the best support based on Δ AICc values. Statistical analyses were performed using R, version 2.15.2, 2012 (Vienna, Austria, http://www.R-project.org).

## Results

### Habitat and bill size Hypothesis 1

Both male and female YEWA restricted to mangrove habitats had larger bills and longer tarsus lengths than their North America inland counterparts (Table 1). Bill surface area was greater in YEWA in mangrove habitat compared with YEWA in terrestrial habitat in both sexes. Tarsus length was also longer in YEWA in mangroves than YEWA in terrestrial habitats.

**Figure 1:**
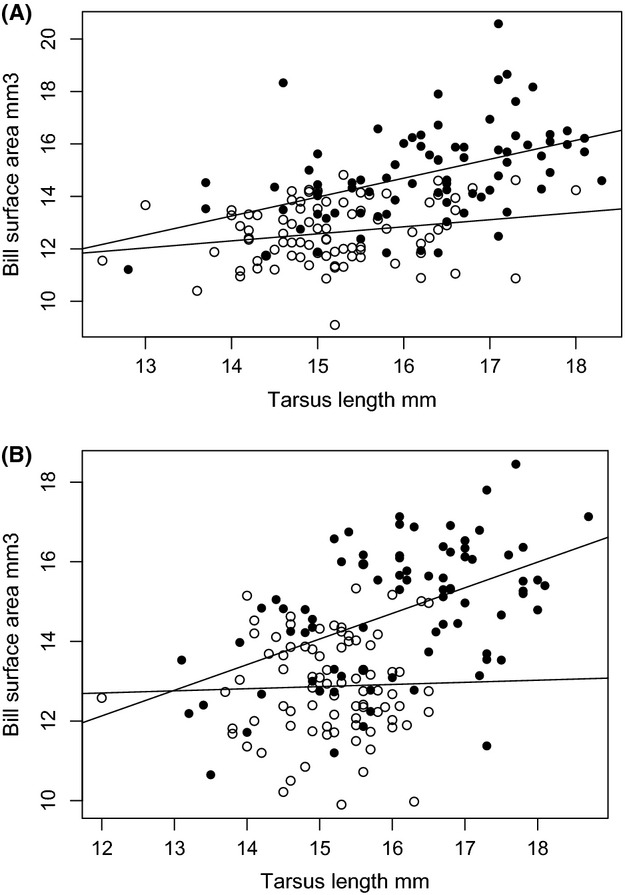
Bill surface area for male (A) and female (B) yellow warblers increases more rapidly with increases in tarsus length for mangrove-restricted subspecies (closed circles and top line) than for inland subspecies (open circles and bottom line). The lines represent best-fit linear regressions.

Results indicate that YEWA in mangroves have bigger bills than YEWA in inland terrestrial habitats (Fig. 1, Table 2) and that bill size increases with mean temperature during the breeding season. The best overall model that predicted bill size was bill surface area = temperature + habitat (model weight 0.71). All other models had Δ AICc values of >2 and weights of 0.21 or less. The global model, which also included habitat × sex, was the second best model, but had a Δ AICc value of 2.47 and weight of 0.21 (Table 3).

**Table 2:** Bill and tarsus measurements for yellow warblers. All measurements are mean values in millimeters with standard error.

Trait	Mangrove		Inland	
	Male	Female	Male	Female
Bill Length	8.33 ± 0.053	8.21 ± 0.067	7.60 ± 0.045	7.63 ± 0.047
Bill Width	3.71 ± 0.037	3.75 ± 0.037	3.51 ± 0.032	3.55 ± 0.029
Bill Depth	3.40 ± 0.032	3.44 ± 0.024	3.15 ± 0.022	3.22 ± 0.021
Bill Surface Area	14.85 ± 0.198	14.78 ± 0.186	12.65 ± 0.112	12.95 ± 0.128
Tarsus Length	16.24 ± 0.125	16.11 ± 0.142	15.18 ± 0.092	15.11 ± 0.079

**Table 3:** AICc models for overall bill morphology.

Model	logL	*k*	AICc	ΔAICc	Weight
Temp + Habitat + Tarsus	−566.11	5	1142.41	0.00	0.71
Temp + Sex × Habitat + Tarsus	−565.27	7	1144.89	2.48	0.21
Habitat + Tarsus	−569.50	4	1147.13	4.72	0.07
Sex × Habitat + Tarsus	−568.74	6	1149.74	7.33	0.02
Temp + Tarsus	−601.71	4	1211.55	69.14	0.00
Temp + Sex + Tarsus	−601.24	5	1212.68	70.27	0.00
Null Model (Tarsus)	−606.86	4	1217.75	75.34	0.00
Sex + Tarsus	−606.44	4	1221.00	78.59	0.00

### Sexual dimorphism – Hypothesis 2

**Figure 2:**
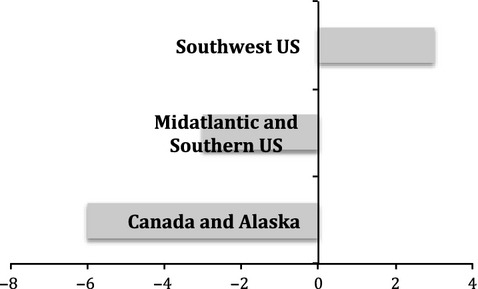
Female yellow warblers have a larger bill surface area (negative values) than males in all geographical regions of the terrestrial subspecies except the southwestern USA.

**Figure 3:**
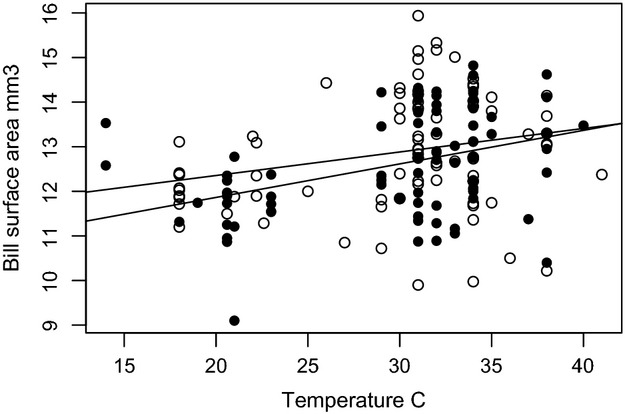
Bill surface area (mm^3^) for terrestrial yellow warbler populations increases more rapidly with increases in mean maximum July temperature for males (closed circles and bottom line) than females (open circles and top line).

Sexual dimorphism in bill morphology was observed among YEWA in terrestrial habitats, but not YEWA in mangrove habitats. In terrestrial habitats, females have larger bills than males, specifically bill depth was larger in females than males (*t*-ratio = 2.69, df = 189, *P* = 0.008; Bonferroni *P* = 0.012). The percent of sexual dimorphism in bill surface area was further divided into each of three terrestrial regions based on subspecies, Mid-Atlantic and southeastern USA (*D. p. aestiva*), southwestern USA (*D.p. sonarana*), and Alaska and western Canada (*D.p. rubignos, amnicola,* and *banksi*). The percent dimorphism ranged from −3 to 6%. Females in populations in the eastern USA and Alaska/Canada had larger bill surface areas than males, but in the southwestern USA, males had bills with larger surface area than females (Fig. 2). Male bill size seems to be more responsive to temperature, especially at the hotter temperatures although the overall slopes of males and females bills in response to ambient temperature are not statistically different (Fig. 3).

### Climate and bill size – Hypothesis 3

There is a positive relationship between bill size and ambient temperature in July for both male and female YEWA in inland terrestrial habitats (linear regression; adjusted *R*_2_ = 0.90). However, in mangrove forests, the relationship between ambient temperature and bill size is not as straightforward with tropical populations of YEWA having no relationship between temperature and bill surface area.

### Mainland and island populations – Hypothesis 4

Region was not an important variable in predicting the bill surface area for YEWA restricted to mangroves in Central America and the Caribbean Islands. There were no differences in bill size between male YEWA restricted to mangroves on islands and on the mainland of Central and South America. The bill surface area of females restricted to mangroves did not differ between inland and mainland populations. Hypothesis 4 was supported, such that birds on islands and in mangroves had no differences in bill size. The best model included only the factor temperature, with a weight of 0.999. All other models had Δ AICc value of 1181 or higher and weights of <0.0001.

## Discussion

While traditional research on the factors that influence bill morphology has been largely one-dimensional, this article highlights the importance of multiple variables in explaining the evolution of bill morphology. We found that bill morphology was best explained by ambient temperature and habitat type, with birds in hotter temperatures and birds in mangroves having larger bills than birds in cooler climates and inland terrestrial habitats. Our results align with previous findings that passerines dependent upon coastal saline habitats (mangroves and salt marshes) have disproportionately larger bills than their closest inland relatives (Grenier and Greenberg 2005; Greenberg and Olsen 2010; Luther and Greenberg 2011). Finally, the correlation between bill size and ambient temperature for inland populations of YEWA extends the previous research on the relationship between bill size and ambient temperature from birds with relatively large bill to body size ratios, such as sparrows (Greenberg et al. 2012a,b), to birds with a relatively small bill to body size ratio, such as warblers. Together these findings are quite novel as they emphasize multiple environmental factors as important contributors toward the evolution of bill morphology.

Contrary to our predictions, we found no sexual dimorphism in bill morphology in mangrove birds, but did find sexual dimorphism in bill morphology among migratory inland subspecies, with male bill size increasing more sharply with high summer temperature than female bill size. Our hypothesis was based on the correlation between sexual dimorphism of bill morphology and high population density found in salt marsh-restricted sparrows (Greenberg and Olsen 2010); however, unlike the salt marsh sparrows, the mangrove-restricted subspecies of YEWA show no sexual dimorphism in bill shape and have lower population densities than their inland sister taxa, which inhabit temperate climes (Lowther et al. 1999; Salgado-Ortiz et al. 2008). The presence of dimorphism in the inland populations does not necessarily contradict the hypothesis that dimorphism will evolve where intraspecific competition is high. The riparian habitat that leftacterizes the temperate mainland populations of YEWA has two qualities, low species diversity and high local abundance of YEWA, that might promote increased intraspecific competition (Lowther et al. 1999). These early succession riparian habitats may have the islandlike qualities that promote dimorphism in ways that are similar to what has been proposed for continental tidal marsh habitats (Greenberg and Olsen 2010). The increased population density of YEWA and larger proportion of sexual dimorphism in bill size in inland YEWA is consistent with the prediction that population density is correlated with sexual dimorphism (Stamps et al. 1997).

Sexual dimorphism in bill size has been documented in a wide variety of species and is generally based on intraspecific competition (Selander 1966; Temeles et al. 2000). In general, males have larger bills than females when there is sexual dimorphism in bill size, except in shorebirds (Szekely et al. 2004). In the few known instances in which passerine females have disproportionately larger or longer bills than males, reversed sexual dimorphism, the dimorphism is thought to be from competition for food resources (Grant and Grant 2003). For example, in some species of hummingbirds and the warbler finch of the Galapagos Islands, females have longer bills than males and it is thought that the reversed sexual dimorphism is a result of male dominance over females at nectar sources; thus, females have longer bills to exploit a wider variety of nectar resources (Bleiweiss 1999, 2001; Grant and Grant 2003; Szekely et al. 2004).

There was great variation in bill shape and sexual dimorphism among the different subspecies of YEWA that inhabit inland habitats (see Fig. 2). Most notably, female YEWA have larger bills than males in the northern and southeastern regions of North America, but males have disproportionately larger bills than females in the southwestern region of North America. The reversal in bill size between sexes and populations could be related to the temperature and/or aridity of the desert climate compared with the northern and southeastern regions of North America. Avian bills can shed large amounts of metabolic heat in hot climates and a larger bill can be advantageous in such climates (Tattersall et al. 2009; Greenberg et al. 2012b). Male birds tend to spend a disproportionate amount of time singing from exposed perches to attract mates and defend territories, while females can often remain undercover, near nests, and in the interior of bushes and trees. Greenberg and Danner (2013) found that the bill morphology male song sparrows in the Channel Islands off of California were more responsive to temperature change than females in the same populations. Similarly, male YEWA might have larger bills than females in the southwestern USA to help release heat through their bills and maintain their body temperature, which could be needed due to increased sun exposure, compared to females, in a hot dry climate.

Recent studies have demonstrated the importance of bills for thermal regulation and a correlation between ambient temperature and bill size (Tattersall et al. 2009; Greenberg et al. 2012a,b). Our results extend the correlation between bill size and ambient temperature previously described (Greenberg et al. 2012a,b) to a relatively small-billed insectivore species, which signifies a more widespread relationship between bill size and ambient temperature than previously documented. Interestingly, only the inland terrestrial subspecies exhibit the strong positive relationship between bill size and ambient temperature, while the tropical mangrove-restricted subspecies demonstrate no relationship. The mangrove-restricted YEWA live in a very narrow temperature range, which is mostly stable, which might explain the lack of a relationship between bill size and temperature. Because of the lack of wide variation in ambient temperature between the different mangrove-restricted populations of YEWA climate might not be a selective force differentially acting on bill size in different populations. Greenberg et al. (2012a,b) found a very strong correlation between bill size and maximum summer ambient temperature among salt marsh-restricted sparrows, *R*^2^ = 0.82, while the inland terrestrial YEWA tested here had a much weaker relationship between the two variables *R*^2^ = 0.16. The difference between the two studies might be attributed to the relatively small bill size compared with the body size of the YEWA compared with sparrows.

Until recently, disproportionately larger bills between closely related populations were thought to only occur in island populations as part of the island syndrome (Clegg and Owens 2002). Recent studies have observed that passerine birds in coastal estuarine habitats, salt marshes, and mangroves also exhibit disproportionately larger bills when compared with close inland relatives (Grenier and Greenberg 2005; Greenberg and Olsen 2010; Luther and Greenberg 2011). We found no difference in bill size or body size between YEWA subspecies dependent upon mangrove forests in mainland Central America and those found in mangrove forests on Caribbean Islands. Our comparison of bill morphology in birds in mangroves and oceanic islands provides direct support of the island syndrome in a mainland ecosystem.

In summary, our study supports multiple factors and hypotheses that correlate with bill size such as ambient temperature, at the breeding site, and habitat type as the most important variables in our models. Surprisingly, we found that ambient temperature was influential on the amount of sexual dimorphism for temperate terrestrial populations but not tropical populations. Finally, we provide new evidence that ambient temperature affects bill morphology in relatively small-billed birds such as warblers, not just in larger bill birds and that future research on the evolution of bill morphology will need to account for multiple factors to explain bill morphology.
